# The Value of Researcher Reflexivity in the Coproduction of Public Policy: A Practical Perspective

**DOI:** 10.1007/s10912-024-09853-1

**Published:** 2024-06-24

**Authors:** Yamini Cinamon Nair, Mark Fabian

**Affiliations:** 1 Greater London Authority, London, UK; 2 University of Warwick, Coventry, UK

**Keywords:** Coproduction, Public policy, Reflexivity, Positionality, Critical theory

## Abstract

Coproduction of public policy involves bringing together technical experts, practitioners, and people with lived experience of that policy to collaboratively and deliberatively codesign it. Coproduction can leverage different ways of knowing and evaluative perspectives on a policy area to enhance the legitimacy and efficaciousness of policymaking. This article argues that researcher reflexivity is crucial for getting the most out of coproduction ethically and epistemically. By reflecting on our positionality, habitus, and biases, we can gain new insights into how we affect the research design, production and analysis of data, and communication of findings. This reflexivity helps to disrupt power dynamics that underly research and policymaking, helping to realise the radical potential of coproduction to democratise practice, empower citizens, and make research more relational. We demonstrate the value of reflexivity through an analysis of our work coproducing a theory of thriving in financial hardship in partnership with the UK national anti-poverty charity Turn2us. We contextualise our advocacy for reflexivity within the practical realities of advancing coproduction in the UK today.

## Introduction

Coproduction is a form of participatory knowledge production that brings together researchers, practitioners, and people with lived experience to collaboratively design policy (Turnhout et al. 2020, Bovaird 2007, Mitlin 2008, Farr 2018). Participatory research realigns the role of the researched into an active participant in the production of data, where participants should have at least some control over the research process even if executive control remains with the researcher (Barber et al. 2012, Bennett & Roberts 2004). Coproduction sits towards the radical end of the participatory research continuum, characterised by a blurring of definitional boundaries between researcher and researched (Frankham 2009). Participatory research, including coproduction, is growing increasingly common in the development, implementation, and management of public policy. The epistemic and ethical case for participatory research is pertinent in policy areas where a lack of understanding and stigmatisation of service users prevail, notably poverty, disability, and welfare (Booth 2019, Osinski 2021, Patrick 2020). In its best form, coproduction offers a potentially radical way to integrate different ways of knowing and lived experience, bringing representation to marginalised individuals and communities that would traditionally be bypassed in the policymaking process.

In this paper, we interrogate the ability of coproduction to achieve these goals, building on others who are concerned that the existing literature and practices of coproduction do not pay enough attention to the role of power in shaping the process and outcomes given the systemic issues coproduction tries to resolve, particularly with the dissolution of the traditional dichotomy of the researcher and researched (Turnhout et al. 2020, Forester 1999). Drawing on Habermasian theories of the public sphere, we argue that researchers and policy professionals engaging in coproduction are active participants in a highly value-laden process of communicative rationality, not neutral bystanders (Habermas 1979). This makes reflexivity a relevant consideration for anyone involved in the milieu of theory coproduction, notably medical humanists and charity organisations operating in contested normative domains like public health and poverty, especially the social determinants thereof (Saffran 2014).

Our analysis contributes to the literature on ‘critical’ medical humanities, especially the work of Viney et al. (2015). They emphasise the potential of a critical research practice ‘based on notions of entanglement, rather than servility or antagonism’ to enhance the imagine, creative, and heterodox capacity of research. These capacities are central to good coproduction aimed at collaboratively articulating, rather than merely uncovering, desired changes to public policies that are socially embedded. They are also central to ensuring that the medical humanities and public voice therein is not coopted by the language and goals of medical science (Wear 1992).

We identify de-politicisation and romanticisation of the coproduction process as a major and frequent pitfall, including in our own work (Fabian et al. 2023a). We apply foundational insights from critical theory, which emphasise the ways in which knowledge is embedded in power relations (e.g. Foucault 1991), to better appreciate how a more reflexive approach of the researcher can enable a more grounded understanding of the power structures coproduction tries to address.

This reflection necessitates a wider recognition of how our ways of knowing are intertwined with our lived experience, our protected characteristics, internal dispositions, and our emotive relatability and intuition beyond reason and rationality. Bourdieu’s concept of the habitus as the ‘structuring structure’ that organises the ‘practices and perceptions of practices’ through which the researcher understands the world is essential to this reflexive practice (Bourdieu 1984, 170). By recognising the positionality of researchers and their active interpretative involvement through their own ways of knowing and lived experience as part of the coproduction process itself, better ethical and epistemic outcomes can be reached (Haraway 1988, 1989; Clifford & Marcus 1986). This is a step towards more conscious research, with academics aware of the power structures we work within and our responsibility to use participatory platforms to advocate for change rather than overpromise radical outcomes (see Patrick 2020).

Such an appreciation of inherent and humane subjectivities within coproduction does not undermine but in fact strengthens its validity, given the relevance of community and identity to the domains of public policy commonly addressed, and the need for subjective value judgements to conceptualise the objectives, metrics, and evaluation of public policy in such domains. Coproduction can be understood as a way to create *public* values that represent collective ideals, aspirations, and appraisals (Nabatchi 2012). We advocate for a radically reflexive acknowledgement of researchers’ implication in existing power dynamics within and beyond the coproduction space to better ensure its products are genuinely representative of collective value and grounded in the structures it addresses.

We illustrate our arguments through an honest reflection on our work coproducing a theory of thriving with the UK national anti-poverty charity Turn2us and lived experts of financial hardship. The theory is now informing the charity’s work, including internal processes and outward-facing campaigns, such as system-change efforts in the UK’s social policy space. The project was grounded in a theory of coproduction that emerged out of debates around responsible social science with value-laden concepts (reported in Alexandrova & Fabian 2022). This is quite a different angle than the more explicitly political agenda of other coproduction traditions, notably critical participatory action research (Sandwick et al. 2018). Notably, our method is principally motivated by epistemic concerns and is not pre-committed to the problematisation of the status quo. Whilst we hope that our method is useful for identifying policy reforms opportunities, we also envision it being institutionalised in policymaking, whereupon an iteration of it might simply find that policy is broadly working well. A paper summarising the process and outputs of our work with Turn2us in detail is available in Fabian et al. (2023a). In a third paper, we analysed the role coproduction and other participatory approaches might play in complementing more prevalent top-down approaches to wellbeing public policy (see Fabian et al. 2023b). We have adapted these three papers into a methods paper for the What Works Centre for Wellbeing discussing the coproduction of wellbeing public policy for a practitioner audience (see Fabian & Alexandrova 2022).

This paper provides a critical appraisal of the role researcher reflexivity, or lack thereof, played in the outcomes of our research. We avoided this undertaking in previous papers in part because we feared that our primary intended audience — UK policymakers and adjacent academics — would be antipathic to talk of subjectivity in research and non-positivist epistemologies. However, following our own unusual collaboration across disciplines, we now recognise the relevance and vitality of acknowledging these inherent subjectivities we carry to improve the ethical and epistemic qualities of coproduction in policymaking. One of us has a background in economic policymaking and (post) positivist paradigms, and the other in sociology and non-positivist research practices. We differ in our ethnicity, gender, institutional and cultural capital, identification with disability, and to some extent our political values. We hope to convey some of the learning that can develop from such contentious collaboration. Whilst the experience can be challenging and uncomfortable, it can also foster learning and appreciation of the research practice as revealing as much about the researcher as the topic of research. We acknowledge a tension that unfolded between the ideals of radical coproduction and getting coproduction meaningfully implemented in contemporary policy environments and contextualise our analysis within these practical realities. We call for a more reflexive approach to coproduced public policy to improve its practical outcomes.

### What we did with Turn2us

**Figure 1:**
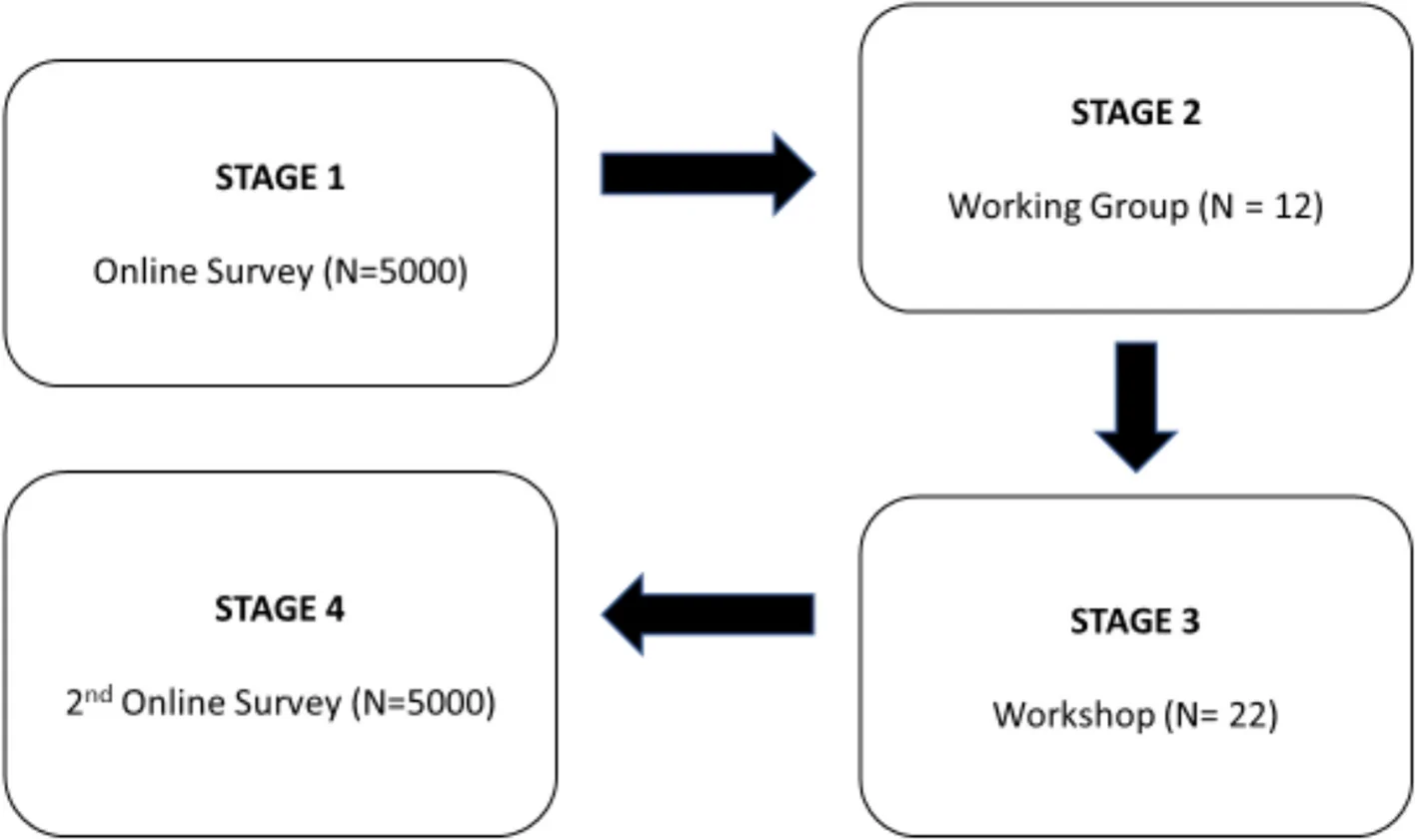
Our coproduction process (see Fabian et al. 2023a for an extensive discussion)

To facilitate the later discussion, we need to briefly sketch here the methodology we employed in our work with Turn2us. Our coproduction methodology consisted of four stages, outlined in Figure 1. The core qualitative component was book-ended by quantitative methods. This promoted a balance of rich, diverse insights and representativeness. We began with a survey of Turn2us service users administered through the charity’s newsletter, which reaches 5000 people. We obtained 1500 responses to five questions, some ranked-choice others short answer, that aimed to get an initial picture of how the people Turn2us works with understand thriving in their lives.

We then constituted a ‘working group’ of 12 individuals — three academics, four people with lived experience, and five staff from across Turn2us, including one director and the charity’s coproduction coordinator. Members of this working group interviewed each other one-on-one over several months to generate data on what thriving in financial hardship means in the context of Turn2us’ work. Two of the academics also interviewed additional staff from Turn2us to get a wide-ranging understanding of the charity’s activities and the role a theory of thriving might play in them. This data was initially analysed by the academics and reported back to the working group in all-group meetings. In these, the analysis of the academics was subjected to questioning, critique, and refinement.

This process of small and large meetings was iterated until the working group had a theory of thriving that it was satisfied with, which was drafted up into a report by the academics. The report was cleared by the working group before being taken to a workshop. Here, 10 additional people with lived experience were invited to read, analyse, critique, and offer improvements to the report.

Once they were satisfied with it, a draft report was published online and recipients of the Turn2us newsletter were invited to evaluate and comment on it through Likert-scale and short answer questions. The reception was overwhelmingly positive, and the report (Turn2us 2022) thus became official Turn2us policy.

## Critical reflections on our coproduction process

### Origins and outcomes

Given coproduction is an attempt to move towards genuine power-sharing to challenge how and why we know what we know, the process of topic consolidation, methodology, and acquiring site access is critical, yet often overlooked. Deciding on the research topic and the field site is mediated by many characteristics, networks, and institutional frameworks. Interestingly, our project began somewhat fortuitously as Turn2us approached us whilst we were at Cambridge University about a separate quantitative analysis on their benefits calculator tool. From that conversation, two academics realised there was a potential partnership to do our research on coproducing context-sensitive theories of wellbeing. Though impossible to be certain, it is worth acknowledging the institutional and symbolic capital Cambridge University holds as an elite and prestigious research university and the role this may have played in gaining access to collaboration with the charity and their lived experts (Khan 2011, Ostrander 1993, Bourdieu 1984).

Coproduction was later introduced at the stage of data collection following a decision by the practitioners at Turn2us regarding what the project should focus on and a decision by the academics as to the process that should be followed. The lived experts of financial hardship, sought by coproduction specialists at Turn2us, were empowered to shape an organisation whose activities they cared about following their own period of hardship and the help they received from Turn2us. Each group — the professionals, the academics, and the lived experts — all had their own decision-making power within their specialist realm. However, upon reflection, we would like to caution the over-simplification of this power-sharing ideal. Notably, whilst the lived experts were empowered and compensated for their time^1^, these benefits were arguably not commensurate with those accruing to Turn2us and the academics.

There are at least two clear examples of how unequal power dynamics were present from the onset between professionals, academics, and lived experts, and how different interests affected the research output. First, the first three academic outputs of the project (Alexandrova & Fabian 2022, Fabian et al. 2023a, 2023b) were written to appeal to a particular audience, one dominated by happiness economists, analytical philosophers, policymakers, and other communities that subscribe to broadly positivist paradigms and are uneasy about critical theory. As such, we strategically sequestered critical and interpretivist perspectives on our work to the present paper and were judicious in avoiding ‘radicalism’ in our descriptions of the project and its intentions (Wagernaar 2012).

Second, the final report of the working group was written for the general public and appropriately tailored, on advice from lived experts in the working group, in terms of visual style, interview quotes, language, and an accompanying visual-storytelling video. The tropes of critical theory and interpretivism were again absent, though this time for reasons of accessibility. We think this sort of pluralism is desirable for the purposes of increasing impact and incorporating different interests and perspectives in research outputs. However, we think the origins of this diversity should ideally be explicitly articulated throughout the project, something we fell short of.

Our experience has strengthened our concerns that coproduction can be used in a tokenistic or tick-box fashion if lived experts are not involved from the beginning of the research, undermining coproduction’s radical potential. An honest account at the outset of the interests of the various parties is important to the success of coproduction, and where ultimate decision-making lies must be interrogated throughout. For example, following the scoping meetings between Turn2us and us as the research team, we would occasionally discuss with interview partners how their research fits into their broader field, and what they hoped this project might contribute to that milieu. Whilst valid, more space could have been created for critical reflection of the fact we were implicated in the research as much as the lived experts.

### Interview process

In a more considered attempt to facilitate genuine power-sharing between the researcher and the researched under the ideal of ‘research with people, not on them’, we adopted a bi-directional interviewing approach following Fujii’s (2017) technique of ‘relational interviewing’ within the working group. This seeks to go beyond the idea of rapport towards a working relationship built on humanistic values of dignity and respecting different ways of knowing. Whilst these efforts were sincerely adopted and quite successful by our estimation, we highlight several limitations.

As the facilitators of this process, we could not provide a neutral space through which lived experts and professional experts could engage and unload their own conception of wellbeing. This is inevitable as there is no apolitical or objective space — institutionally, socially, emotionally — where views can simply be ‘uncovered’ (Orr & Bennett 2009). Instead, the responses people gave — verbally and non-verbally — were mediated by many factors ranging from life experiences linked to their upbringing, educational background, networks of friends and family, protected characteristics such as ethnicity and disability, individual mannerisms, body language and emotive and affective communication, and how these related or did not relate to the person they were talking to. These need to be recognised to understand how the data produced is a product of these circumstances and thereby move towards more context- and time-specific policy. Coproduction as a method does not engage directly with existing knowledge-power dynamics, addressing *how* each participant knows what they know. Instead, it simply attempts to bring these situated knowledges to the same table.

Bourdieu’s (1984) concept of the habitus and individual dispositions can further be applied to the interview process to better understand knowledge-power dynamics. What we decide to uncover, or hide, during the interview process as active participants mediates how we interact with others and the data that is subsequently generated. For example, in Rivera’s 2015 ethnography of elite students, she positions her own multi-faceted identity or mixed habitus (Friedman 2016), background, and experiences as assets in conducting research and her ability to ‘character switch’ between her working-class upbringing and her experience attending elite education. As a result of this juxtaposition, she states she was able to connect with interviewees on each occasion and obtain a richer picture of their experience through a shared institutional cultural capital and human to human understanding. She says that ‘*without a sense of commonality, I doubt my interviewees would have been comfortable disclosing much of the sensitive data they did*’ (Rivera 2015, 25).

We were similarly active participants as interviewers and interviewees in the research, shaping the formation of the data. We were able to ‘character switch’ according to what was visible versus invisible, and our feelings and thoughts at the time. Our ethnic and gender identities were visible but our identities as a disabled or queer person can be hidden, especially given their degree of invisibility within wider society. We disclosed these hidden identities at certain times and not others. For one of us, the interviews coincided with the early stages of a medical diagnosis and dealing with acute physical pain, which alongside the unique time of the pandemic, led to a focus on health and perceptions of health as fundamental to wellbeing. This may not have been the case if the interviews took place today. Relatedly, one of us identifies as non-disabled and at this time had not encountered academic or social justice work around the disabled movement and the social model of disability (see Oliver 2012). This led to a knowledge exchange with a lived expert with a background of activism around disability rights that served not only to equalise power dynamics but reverse them in this specific setting. This is an example of power-sharing through the exchange of situated knowledge with our relational and humanist approach, and of the research as a process of becoming.

One of our key learnings from this project is how coproduction requires a high level of emotional awareness, vulnerability, and consciousness of ourselves as human beings beyond our academic roles. In the present transition among academics from research ‘on’ to research ‘with’ people, such considered reflexivity remains relatively unconventional. The more natural ability of lived experts to connect their wider habitus, characteristics, and life experience, with how they see the world and ascribe values within it starkly contrasted with the concerted effort required by us to make similar connections.

### Analysis process

The analysis of interviews was conducted by two of the three academics in the working group (the authors of this paper), who also participated in the interviews and coded each other’s interviews. One researcher had a substantial academic background in wellbeing theory whilst the second researcher did not. In an attempt to harmonise these two perspectives, the first researcher used a deductive approach to thematic analysis, grounded in existing theories of wellbeing, whilst the second used an inductive approach, bringing less theoretical bias at the coding stage (Braun & Clark 2006). This attempt to leverage different academic backgrounds and ways of knowing (induction versus deduction) is a constructive example of incorporating researcher reflexivity into methodology. It served to place the researchers as active participants within the analysis of the data as well as the data collection. The recognition that knowledge is situated worked to strengthen the methodological rigour of the work and we were reassured that the analytical codes each researcher produced were broadly comparable (see Fabian et al. 2023a).

However, our efforts to acknowledge how our positionality impacted the analysis process could have been more radically reflexive. The risk of differentiating deductive and inductive approaches to thematic analysis is that this becomes a naïvely positivist approach in and of itself, where the emergent human coder is merely ‘unearthing’ the truth rather than bringing their own subjectivities and biases to the analysis. Whilst constrained by time and resources, more space was needed to reflect on the characteristics and lived experiences of the researchers and how the analysis is a product of ourselves. In this sense, we are not merely implicated in the research, as the interview process rendered, but the product is again a process of our own becoming.

For example, one analyst has a well-publicised view of what wellbeing is (Fabian 2022). This view is antithetical to the perspective in hedonic psychology and happiness economics that wellbeing is defined by experienced moods and evaluations of life satisfaction. As such, it is possible that the observation from the qualitative data analysis that ‘happiness’ did not seem to figure in thriving in financial hardship was a product more of this researcher’s biases than the raw data itself. Recognising this positionality, we made the raw data from our project publicly available to any researchers who wanted to conduct their own analysis of the data to check whether their interpretation differs. However, because of the power this researcher had as project manager and an active participant in the data-generating process, it is possible that the raw data itself is suffuse with their biases. In this case, simply making that data available for cross-checking does not address whether the project itself is biased.

### Interpretation

The process of transforming text data into interpretation and discussion always warrants many considerations, but we focus here on three that are especially relevant to this critical reflection.

First, the analysis was conducted by two researchers rather than co-analysed by the working group. Whilst lived experts in particular were encouraged to speak up and generate data in the interviews, the analysis and therefore value attribution given to their words was not coproduced. However, importantly in our project, this was partly due to our coproduction partners resisting involvement in this process. As we outline in Fabian et al. (2023a), the lived experts did not want to come up with their own interview questions and actively requested scripts and guidance on several occasions. They did not want to participate in the data coding, mostly for reasons of having insufficient time and training to analyse around 80,000 words of transcript data. They also did not want to lead in organising the workshop but were happy to chair discussions in breakout rooms provided they were given some guidance and a rough script for how to do so. All three of these tasks were beyond what the coproduction partners had volunteered for. In many ways, these are onerous tasks that require extensive labour. The partners were instead happy to contribute their respective strength — namely lived experience — and provide oversight on the coproduction process by participating in it. One lived expert even chose to write a poem to describe what thriving meant to her, which we included in the Turn2us report, reminding us as academics of the importance of making space for artistic expression as a rich form of emotive, intuitive, and narrational data. These cases demonstrate the agency of the lived experts in the analysis process of this project.

Some coproduction scholars might argue that the lack of co-analysis of raw data in our project disqualifies it from being coproduction. We strongly disagree. Our methodology upholds the five principles of coproduction outlined by Hickey et al. (2018). It involves substantively more power-sharing and two-way learning than consultation. This is evident from our surveys of participant sentiment throughout the process (newsletter, working group, and workshop), wherein they said that they felt heard, and the outputs reflected their own thoughts and feelings. We lost sleep over the possibility that the coproduction partners in the workshop and final survey would reject our framework for thriving in financial hardship as it would have required us to start the coproduction process over again. It is difficult to observe the sincerity of this sentiment. Some academic practices aimed at checking for it, like formulaic positionality or humility statements, can lead to its mere *performance* by unscrupulous researchers. We transparently produced the (very positive) results of our surveys in appendices to Fabian (2023a), and the raw data is publicly available. There is also a clear and meaningful degree of co*production* in our project*.* Lived experts were not studied for their views on thriving as in a focus group. They were instead part of a long deliberative process that created a theory of thriving drawing on diverse perspectives beyond their own. Our methodology leverages the comparative advantages of the three expert groups — technical expertise from academics for rigour, hands-on knowledge from practitioners for ready implementation, and lived experience from coproduction partners for legitimacy. Far from falling short of coproduction, ours is a sensible, feasible, and efficacious approach to it that is readily adoptable amidst existing policymaking architectures (Nostikasari & Casey 2020).

Second, we need to acknowledge the privileged position of the researchers functioning as mediators between the other participants and the raw data in our project. The data summaries we provided to working group members in our meetings, and to the workshop participants in the form of the draft report, were to some extent a product of our way of knowing, practices, and perceptions of the world — always somewhat governed by our own habitus. We endeavoured to present our summaries as openly as possible, earnestly asking the coproduction partners whether the themes resonated with them and what they wanted changed or adapted. This marks good practice in coproduction, to attempt to share decision-making power as frequently as possible, though acknowledging the unique position of us as academics in data analysis and interpretation. We are aware that our lived experts were not professionally trained qualitative researchers as we are, and though brief training in interviewing techniques was provided, participants felt this was beyond their comfort zone. Acknowledging the different but equal value lived, academic, and technical forms of expertise within coproduction allows for a more reflexive and cooperative approach to the process overall.

**Figure 2:**
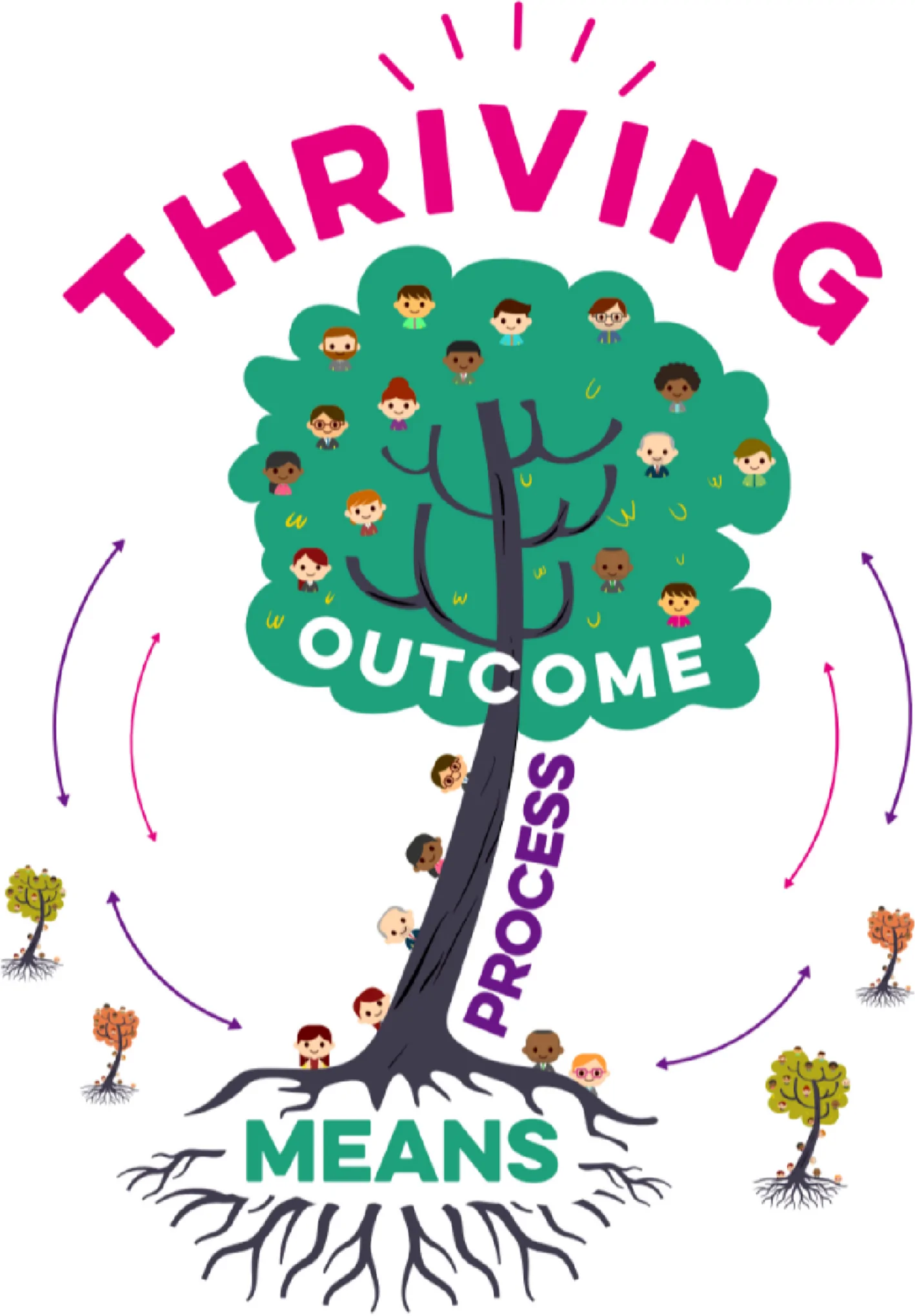
The tree metaphor for thriving in financial hardship

An illustration of this dynamic is the tree metaphor at the centre of the final theory of thriving (see Figure 2). The working group thought it necessary to present some model of our theory that showed how its different components (means, process, and outcomes) were dynamically interrelated. Early efforts at such a model developed by the researchers were rejected by the coproduction partners for being too technical (notably those involving the language of the capabilities approach — Robeyns 2017). The group eventually settled on the tree model in large part because the people with lived experience found it visually accessible, metaphorically appealing, and comprehensive. It is not ideal from an academic point of view because it blends ideas from several wellbeing theories without explaining why they need to be combined. This case illustrates how power and epistemic labour were shared across the working group and also highlights the central position of the academics in the analytic work. Undoubtedly, this affected the outputs of the process given the ultimate discretion afforded to us as the researchers, though this is a good case of bringing together different forms of knowledge for a collective outcome.

Third, the full extent of the researcher’s habitus on the production of meaning from the text data calls for reflexive consideration. One specific example is that in Fabian et al. (2023a), interview text is quoted with the words ‘black’ and ‘disabled’ in lowercase, rather than with the first letter capitalised. Whilst this may seem like a trivial detail, the semantics expose a differing habitus and way of knowing between researchers. The lowercase is used generically whilst uppercase ‘Disabled’ and ‘Black’ carries significant political weight in respective social justice movements (e.g. see Du Bois, 1920, Lorde, 2019/1984). This example also highlights technology as a mediating factor and the subjective decision-making that is taken by a human coder when transcribing audible interview data into text. It further highlights that the power dynamics between members of the research team, whilst collaborative, was dependent on not just differing levels of academic experience and qualifications — institutional cultural capital — but also varying time commitments that changed who held the pen on the final text. The researcher most aware of and invested in the politics capitalisation also had the least institutional cultural capital (no PhD). As a junior on the project and having moved into a non-academic position by the time the paper was finalised, there was also an element of imposter syndrome and concern about who was an ‘insider’ in the academic space versus an ‘outsider’. This is an important reflection to appreciate power imbalances not just within the working group, but within each form of expertise.

## The value of reflexivity in policy coproduction

Participatory approaches to knowledge production — which in theory aims to address the epistemological and ethical dilemmas of policymaking — are becoming increasingly commonplace in public policy (van der Graaf et al., 2021). Whilst this is a deliberate attempt to embrace normativity and make space for multiple actors, there remains little reflection in this space on the active role of the researcher and the impact of our subjectivities on the data. Coproduction efforts often remain rooted in an arguably naïve positivism that sees coproduction as a process of ‘uncovering’ an objective truth rather than an active process of *production*. If participatory research is committed to acknowledging and addressing power imbalances and ultimately improving the lives of our citizens, greater researcher reflexivity is required (Bussu et al., 2022). Our reflections above demonstrate the positive role a more reflexive approach to the research can provide in providing insights into the complexity of power dynamics, different ways of knowing, and their impacts on the output of research produced. Honest and critical reflections of our own research is an important humbling practice that reminds us that we as researchers are part of the subject we are studying and the process by which we are studying it. This removes boundaries between the researcher and researched.

Practically, space can be created to move beyond ubiquitous positivist thinking and embrace inherent subjectivity. By acknowledging the active role of the researcher, we can work towards research as a process of learning, understanding, and improving — rather than simply revealing. This is an essential practice given the increasing use of participatory approaches, necessarily implicating the researcher, in government. There is no perfect research, but there is a more reflective practice that seeks to dissolve imaginative binaries and understand how we are implicated in, and a product of, the overarching ‘structuring structure’. As a starting point, this may take the form of explicit dedication to thinking about our positionality, as von Unger et al. (2022) did with the creation of reflection labs, training sessions, and lunchtime seminars, or establishing staff networks committed to interdisciplinary research. We want to emphasise the goal here is to enrich both the rigour in which coproduction is conducted in policymaking and the researcher’s own critical awareness, moving towards coproduction research as an ever-evolving, dynamic process with more accurate outcomes for policymaking through a more considered understanding of the power, ethics, and epistemic dynamics coproduction tries to address.

## A practical way forward for coproduction policymakers

Having reflected on the value of reflexivity in coproducing public policy, we close by contextualising our analysis against our experiences and discussions amidst the policymaking community (predominantly within the UK and Australia, though with some forays into the global community). A prominent feature of governance in contemporary rich nations is the dominance of quantitative evidence and methods in policy analysis and evaluation, especially in central agencies and among senior officials (OECD 2020). An approach to coproduction that does not at least plug into the way people steeped in quantitative approaches think is unlikely to be favourably received. At the same time, the power of narrative and anecdotal evidence from constituents on the decisions of politicians is well documented in political science (Cairney & Kwiatkowski 2017, Grube 2022), and this sort of evidence is the domain of qualitative methods. This is part of why Alexandrova & Fabian (2022) suggest bookending the core qualitative component of their coproduction method with quantitative methods that provide representativeness. Yet mixed approaches of this sort remain rare in coproduction efforts and advocacy.

Coproduction, especially in the style emerging out of participatory action research and other radical traditions, comes with certain value-judgements embedded. These are anti-technocratic at a minimum, but they can be substantially more radical than that. In our experience, these values are not widely shared among policymakers, especially those of a conservative or strongly positivist persuasion. This raises a quandary for coproduction advocacy: how to increase the use of coproduction without compromising its embedded values? We outline some areas, among many, where this quandary is especially salient. Our intention here is not to pass normative judgement, but rather to contextualise efforts to advance coproduction in public policy that may be considered problematic by theorists. Among policymakers, academia is easily criticised as an esoteric institution, disconnected from the reality of policymaking and the avenues to implement tangible change, constrained by political and policy cycles and the need for financial accountability (Guerin et al., 2018).

Our first area is relatively benign: paying people with lived experience. With few exceptions, this is widely regarded as a minimum ethical standard. But it can be surprisingly complex. In the UK in 2023, the government requires ‘right to work’ checks to be conducted in person using original copies of relevant documents^2^. If the coproduction work is taking place away from the host institutions, such as when it is for a national organisation, these right to work checks can be prohibitively onerous. Besides administrative burden, these and other on-costs associated with adding someone to payroll, such as taxes and HR staff, quickly inflate the cost of coproduction. A common approach in such circumstances is to offer participants shopping vouchers instead of payment. Here too, regulation can cause headaches. In the UK, ‘gifts’ of more than £50 to employees are taxable, which triggers the administrative burdens outlined above. At the current London Living Wage of £11.95 per hour, this puts a cap of four hours on what a coproduction partner can contribute to a project. This is barely sufficient for the workshop that we ran with Turn2us. Some may also object to paying people in vouchers, especially people in complex disadvantage. As social researchers, we were hesitant to provide vouchers given the historical discourse around the ‘deserving’ versus ‘undeserving’ poor. However, we found that coproduction partners in receipt of benefits often preferred vouchers because these do not count as income in benefits calculations.

One consequence of the above complexity is that a ‘gold standard’ coproduction can be expensive. Local governments and community organisations on tight budgets may be reluctant to fund such exercises. A cheaper approach, such as one like ours leveraging the comparative advantages of different expert groups, may be more palatable. Coproduction advocates should be alive to these issues both in their outreach and impact efforts and in peer review — what is ethical may be highly dependent on the circumstances and thus we advocate for a contextually nuanced approach.

A second area we want to flag is how people with lived experience are involved in coproduction. Thorough involvement, by which we mean involvement in all aspects of the project from conceptualisation and coding through to writing and dissemination, whilst often ideal, can sometimes be problematic. The time commitment required makes recruitment challenging. In many contexts, the sorts of people with the capacity to make such a commitment are unlikely to be representative of the population whose lived experience you are trying to centre. Thoroughgoing participation can amount to a part time job, which can compromise the idea that the people with lived experience stand apart from the institution and power structures that research is trying to disrupt. If the coproduction partners depend for their livelihood on the organisation conducting the coproduction, this can undermine their ability to contribute frankly and fearlessly. Training people in the skills required for many aspects of coproduction, such as qualitative coding, is expensive and time-consuming. Whilst it may be ethically desirable, it may add little epistemically beyond what can be learnt from lived experts deliberating over the findings of the data analysis. It is questionable whether these marginal benefits are a worthwhile expenditure of the research budget, especially where the coproduction is a one-off exercise rather than part of an ongoing or institutionalised effort.

We are aware that some scholars may counter that training is straightforward and doable, for example in the growing space of ‘citizen scientists’. But if coproduction partners can do this work with minimal training or oversight by the technical experts, it undermines claims that the technical experts bring value to these exercises. The same is true of claims that the technical experts’ role is merely to give greater voice to the people with lived experience. Academics are essentially outing themselves as redundant here. A countervailing perspective is that putting people with lived experience at the forefront of coproduction like this puts an excessive burden on them. With little training, they are expected to provide cutting insights and articulate solutions to longstanding and complex policy problems. Instead, our process demonstrates the differing values of lived experts, professionals (technical experts), and researchers (academic experts) and evidences the agency of lived experts in deciding the extent to which they would like to participate.

Whilst we advocate for a more reflective, open, and humble approach to the process, we would caution against setting a high and ever-rising bar for what counts as coproduction as this can demoralise well-meaning practitioners. Expectations for extensive training, high wages, and project leadership from coproduction partners coming from outside an organisation with little loyalty to it all discourage coproduction taking off. We would also caution against getting mired in the ethical complexities of coproduction and consequently losing sight of the beneficial outcomes that it is supposed to deliver. There is no shortage of case studies of highly inclusive, procedurally sophisticated, and fair coproductions that produced little of value at great cost. A recent example from Los Angeles is the La Sombrita bus stops. These emerged from an arguably performatively diverse, equitable, and inclusive coproduction process with the high price tag of $200,000. Yet they provide neither the shelter nor safety that the coproduction was ostensibly convened for in the first place (Capps 2023).

We close this section by returning to the theme of policymakers who may be ideologically opposed to some of coproduction’s more radical aspirations. When confronted by such actors, it is advisable to strategically present coproduction as improving governance outcomes rather than ‘challenging’ or ‘disrupting’ it, though this may be its radical intention. Coproduction of the sort outlined by Alexandrova & Fabian (2022) taps local, tacit, and visceral information; empowers citizens whilst remaining in a liberal-democratic paradigm; and devolves decision-making to where policy is delivered. Ultimately, coproduction can enhance governance outcomes by creating place-based policy using bottom-up data with the legitimacy of public buy-in. There is room to acknowledge subjectivity given the value-laden topics coproduction is often used in, without this undermining the quality of the outcome. Embracing subjectivity in policymaking gives the opportunity for more people, power, and place-focused policy. These qualities are all substantial improvements over the dominant technocratic, top-down approach and are thus likely to make coproduction palatable to policymakers.

## Conclusions

Radical researcher reflexivity can enhance the transformative power of coproduction. Reflection on our positionality and the subjectivities we bring to the process is far from a methodological weakness to be minimised, but central to the process. It frequently provides critical insights into underlying power structures and unhelpful assumptions we easily make as researchers. Radical reflexivity requires a recognition that coproduction research is not something that is done to the subject, nor is it a process of unearthing novel discoveries, but is instead a dynamic and relational process where we as researchers are inextricably linked to the production of data. We call for a dissolution of the false boundary between the researcher and the researched in this sort of research for policymaking, and advocate instead for the active embrace of research as a process of becoming and creation. The normative judgements we as researcher-participants constantly make in the coproduction process are a product of our internal dispositions, our habitus and ways of knowing, and understanding of the world. Our interdisciplinary collaboration in authorship of this paper revealed to us the relevance of epistemically opposed disciplines coming together to improve practical outcomes. Policy research is enhanced by the integration of mixed methods, diverse bodies of knowledge, varieties of communication, and competing ideologies. Indeed, this is part of the raison d’etre of the medical humanities, which seeks to bring insights from the expression, creative, subjective, affective, and meaning-making humanities to traditionally biological, chemical, physical, and objective medical sciences. Our reflections illustrate that public policy and public health outcomes can be improved both epistemically and ethically through a more reflexive approach to the practice. We also acknowledge opportunities for collaboration beyond the site of coproduction, and we are keen to explore ways participants can remain involved in the policy influence stages in future projects. A cooperative attitude of this sort can overcome some of the roadblocks to coproduction in contemporary public management and accelerate more effective uptake of the practice.

## Data Availability

No new data was collected or secondary data analysed in the production of this manuscript. All data for Fabian et al. 2023a is publicly available on the second author’s website.

## Endnotes

^1^ People with lived experience in the working group and workshop were paid the London Living Wage for their time.

^2^ Right to work checks were allowed to be conducted digitally during the COVID-19 pandemic.
